# Biomimetic Enzyme Cascade Structural Color Hydrogel Microparticles for Diabetic Wound Healing Management

**DOI:** 10.1002/advs.202206900

**Published:** 2023-03-22

**Authors:** Li Wang, Guopu Chen, Lu Fan, Hanxu Chen, Yuanjin Zhao, Ling Lu, Luoran Shang

**Affiliations:** ^1^ Department of Otolaryngology Head and Neck Surgery Institute of Translational Medicine Nanjing Drum Tower Hospital School of Biological Science and Medical Engineering Southeast University Nanjing 210096 P. R. China; ^2^ Oujiang Laboratory (Zhejiang Lab for Regenerative Medicine, Vision and Brain Health) Wenzhou Institute University of Chinese Academy of Sciences Wenzhou Zhejiang 325001 P. R. China; ^3^ Zhongshan‐Xuhui Hospital, and the Shanghai Key Laboratory of Medical Epigenetics the International Co‐laboratory of Medical Epigenetics and Metabolism Ministry of Science and Technology(Institutes of Biomedical Sciences) Fudan University Shanghai 200030 P. R. China

**Keywords:** antibacterial, hydrogel, inverse opal, reactive oxygen species (ROS), wound healing

## Abstract

Hard‐healing diabetic wound brings burgeoning physical and mental burdens to patients. Current treatment strategies tend to achieve multistage promotion and real‐time reporting to facilitate wound management. Herein, a biomimetic enzyme cascade inverse opal microparticles system for wound healing, which is intergated with glucose oxidase (GOD) and copper peroxide (CP). Such microparticles are composed of biofriendly hyaluronic acid methacryloyl (HAMA) and pH‐responsive acrylic acid (AA), which provided abundant binding sites and spaces for chemical immobilizing and physical doping of enzymes and metal bioinorganics. When the cascade catalytic system is applied on wound sites, hyperglycemia environment would serve as a hydrogen peroxide (H_2_O_2_) generator through GOD catalysis, while acidic environment triggers the decomposition of CP, further catalyzing H_2_O_2_ to generate reactive oxygen species (ROS). Additionally, the distinctive structural color of the microparticles can visually reflect the wound pH and intelligently estimate the healing state. It is demonstrated that such microparticle systems exhibit excellent broad‐spectrum antibacterial and angiogenesis‐promoting properties, as well as significant real‐time reporting ability for wound healing. These features indicate that enzyme cascade structural color microparticles possess valuable potential in wound healing and related field.

## Graphical abstract

Biomimetic enzyme cascade structural color hydrogel microparticles are fabricated by integrating inverse opal with glucose oxidase and copper peroxide nanodots. This composite microparticles construct a new stratagem with multiphase improvement and visual monitoring ability via structural color for hard wound healing, exhibiting excellent potentiality as a novel multifunctional patch in clinic treatment.

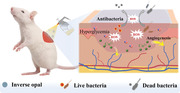

## 1 Introduction

Diabetic wound healing is a chronic and complex process involving four fundamental stages of hemostasis, inflammation, re‐epithelization, and granulation remodeling.^[^ ^1^, ^2^ ^]^ Each stage is vitally important and closely coordinated for follow‐up healing effect.^[^ ^3^, ^4^ ^]^ Hence, a variety of active materials have been presented to overcome obstacles of these stages.^[^ ^5^, ^6^, ^7^, ^8^, ^9^ ^]^ Among the materials, many of them are focused on the generation of reactive oxygen species (ROS) for effectively controlling bacterial infection by employing biological or biomimetic catalysis.^[^ ^10^, ^11^, ^12^, ^13^ ^]^ In addition, it has also been reported that by integrating various metal bioinorganics into these materials, including calcium, zinc, and copper, they have potently worked in the activation of signaling pathways and promotion of cell migration and proliferation.^[^ ^14^, ^15^, ^16^, ^17^, ^18^ ^]^ Despite with many advantages, most of these materials are only aimed at one or two healing stages, leading to poor effects on final healing. Besides, existing biomaterials remain difficult to intelligently monitor the wound condition, which limits their clinical wide‐ranging application. Thus, it is still highly expected to develop a new stratagem with multiphase coverage and monitoring ability for hard wound healing.

**Figure 1:**
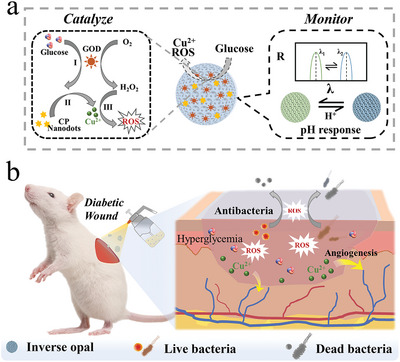
Scheme of the preparation of composite GOD‐immobilized inverse opal microparticles and its cascade reaction in infection of diabetic wound. a) Cascade reaction and monitoring the ability of cascade structural color microparticles. I represents the catalytic process of GOD; II represents the decomposition of CP nanodots; III represents the generation of ROS. b) Scheme of cascade microparticles system applyed in an acute wound model.

Herein, we proposed a novel cascade catalytic hydrogel system by integrating glucose oxidase (GOD) and metal bioinorganics (MBs) into structural color inverse opal particles for multiphase wound healing promotion and microenvironment monitoring, as schemed in **Figure** **1**. GOD, as a scavenger of glucose, could efficiently relieve the hyperglycemia environment and produce antibacterial ROS intermediate.^[^ ^19^ ^]^ In addition, MBs have been proposed as a promising type of nanoagent to combine with GOD, which could form a cascade catalytic reaction and greatly generate ROS,^[^ ^20^, ^21^, ^22^, ^23^ ^]^ exhibiting valuable potential in wound healing.^[^ ^10^, ^24^, ^25^ ^]^ However, simply using GOD and MBs at the injured site is difficult to achieve continual support in multiphase wound healing due to short half‐life and the easy loss of enzymatic activity. In contrast, inverse opal, with interconnected micropores and rich specific surface area, possesses sufficient sites and space to upload and protect bioactives, thus avoiding unnecessary deactivation of protein and low utilization of drugs.^[^ ^26^, ^27^, ^28^, ^29^, ^30^ ^]^ Besides, the feature of its structural color endows the inverse opal system with designable monitoring and sensing ability.^[^ ^31^, ^32^, ^33^, ^34^ ^]^ Therefore, the integration of such cascade catalytic system into the inverse opal would open a new approach for multistage wound healing and real‐time reporting.

In this paper, we developed the desirable inverse opal microparticles with GOD immobilizing and copper peroxide (CP) nanodots doping for diabetic wound healing. The inverse opal microparticle scaffolds were created through photopolymerization of a pregel to reversely replicate spherical colloidal photonic crystals. Such pregel was mainly made‐up of biofriendly hyaluronic acid methacryloyl (HAMA) and pH‐responsive acrylic acid (AA). Through chemical crosslinking, GOD could be immobilized on the scaffold, and the modified microparticles were immersed in a CP nanodots solution to achieve further payload. Once these composite microparticles were applied on the wound site, the hyperglycemia environment could severe as a hydrogen peroxide (H_2_O_2_) generator through GOD catalysis. In addition, benefiting from acidic microenvironment created by GOD catalysis, CP nanodots could decompose to produce copper ions, followed by catalyzing H_2_O_2_ to further generate ROS. Thus, it was proved that the cascade catalytic microparticles exhibited satisfactory antibacterial properties and neovascularization effect in wound healing. Notably, because of their pH‐responsive scaffolds, the distinctive structural colors of the microparticles could visually reflect the wound pH and intelligently estimate the healing state. This kind of patch with multistage wound repair promotion and real‐time monitoring through signal conversion is novel. These results implied the valuable application of the cascade catalytic inverse opal microparticles in clinic wound therapy and related biomedical field.

## 2 Results and Discussion

**Figure 2:**
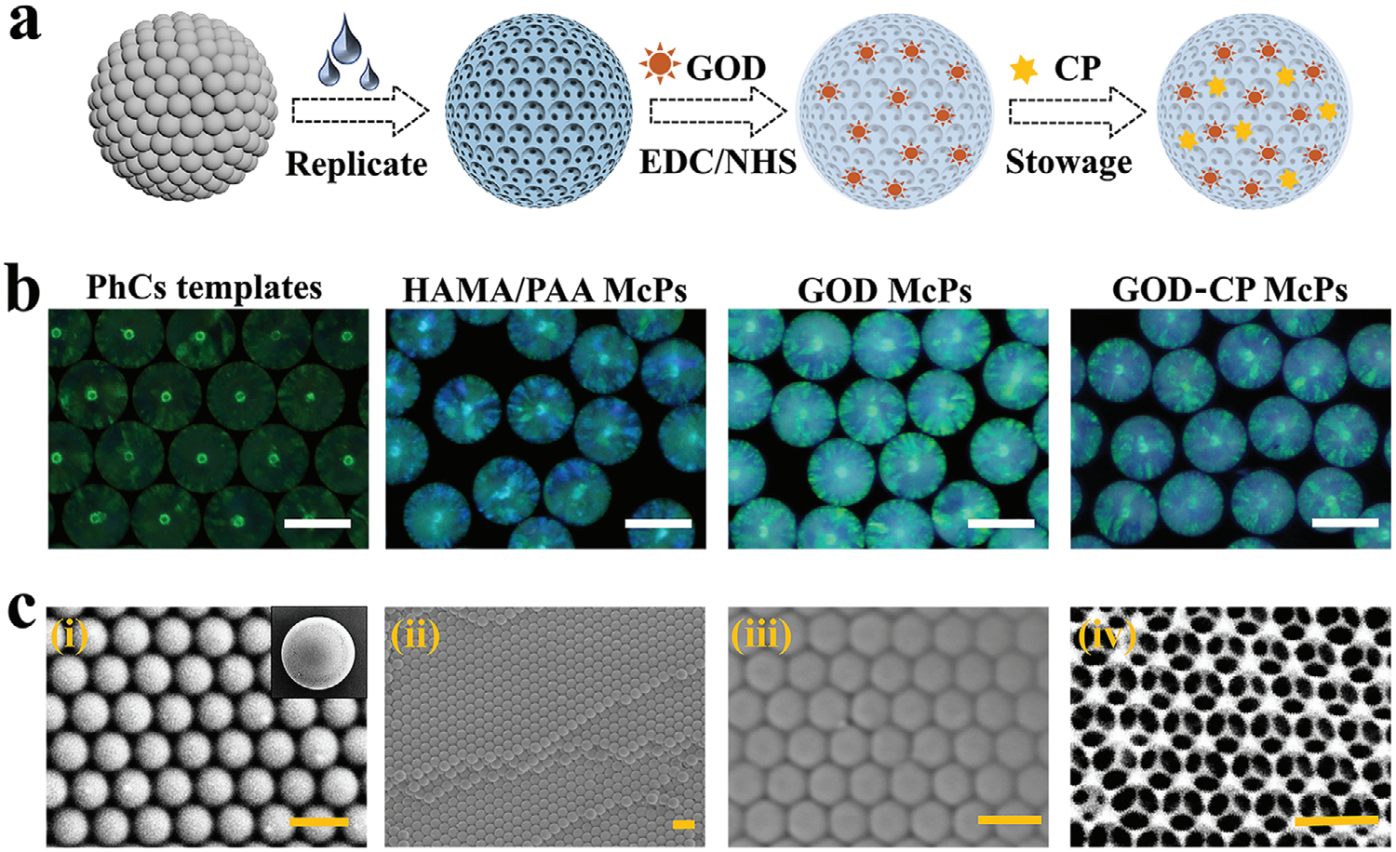
Preparation and characterization of composite GOD‐immobilized inverse opal microparticles. a) Diagram of the preparation of GOD McPs. b) Microscopic images of PhCs templates, HAMA/PAA McPs, GOD McPs, and GOD‐CP McPs. c) SEM images of corresponding McPs. From left to right, the images corresponded to surface structure of PhCs templates (i), inner structure of PhCs templates (ii), hybrid McPs (iii), and inverse opal HAMA/PAA McPs (iv). Scale bars are 300 µm in (b), and 500 nm in (c).

In a typical experiment, monodisperse silica photonic crystals (SPCs) were fabricated by the microfluidic technique with the assistance of a capillary device. Under the action of shear force, a suspension of silica nanoparticles was dispersed into droplets with excellent sphericity and uniform diameter at the T‐junction of the device. After water evaporation and sintering, the silica nanoparticles were arranged and stacked gradually in a specific manner, thereby forming a periodic and highly‐ordered arrangement in the SPCs (**Figure** **2**). Such well‐regulated stackings created interconnected nanochannels regularly existing in the particles. As light passes through, it would be selectively screened by unique photonic band gaps, resulting in a special structural color.

The reflection peak *λ*_0_ of SPCs could be estimated roughly by a simplified Bragg diffraction equation:

(1) $$\lambda_{0}=1.633\;dn_{\mathrm{average}}$$

In this equation, *d* represents center‐to‐center distance between adjacent nano‐units. In addition, *n*_average_ means the average refractive index of microparticles. Based on this equation, microparticles with similar structures could be correctly forecasted. In terms of SPCs, the refractive index of silica is usually constant, and the value of *d* is typically equal to the size of nanoparticle. Thus, the diameter of silica nanoparticle was a main factor to adjust the visual color of SPCs. When the particle size increases, the assembled SPCs would show a red shift in visual color, and the reflection spectrum changes accordingly. Due to the designability and regularity of such structure, SPCs and their derived materials have aroused great interest worldwide, which were broadly applied in many fields such as multicolor printing, ornaments, and signal sensing.

Herein, Figure 2a was illustrated the fabrication of inverse opal based on SPCs and the subsequent modification processes. Intelligent inverse opal was fabricated by reversely replicating SPCs template. Briefly, the dried SPCs were immersed in a pre‐gel solution containing 10% HAMA, 10% acrylic acid (AA), and 1% 2‐hydroxy‐2‐methylpropiophenone (HMPP). Through capillary force, the pre‐gel solution quickly filled the space between adjacent silica nanoparticles, and finally formed a bulk solid after being photo‐polymerized under ultraviolet light. Due to the difference in the external and internal expansion coefficient of the mixed system, the external hydrogel of SPCs templates could be removed easily by stirring or lightly grinding. Subsequently, the templates were etched by 4% hydrofluoric acid to obtain inverse opal hydrogel particles (abbreviated as HAMA/PAA McPs). FT‐IR spectra demonstrated that HAMA/PAA McPs owned abundant carboxyl functional groups which could further achieve GOD immobilization (Figure S1, Supporting Information). After hydrogel microparticles were activated by EDC/NHS, the peak of *σ*_C = O_ remarkably strengthened due to the influence of NHS. Meanwhile, the absorption peak located at 1221 cm^−1^ belonged to the C–N bond. These data indicated that the HAMA/PAA carriers were successfully activated and could further be applied to fix GOD.

In the experiment, microparticles at different stages showed varied colors attributing to changed material components and structures. The images of corresponding microparticles were recorded by optical microscope in Figure 2b, including SPCs templates, HAMA/PAA McPs, HAMA/PAA McPs with GOD immobilization (GOD McPs), and HAMA/PAA McPs with GOD immobilization as well as polyvinyl pyrrolidone coated CP loading (GOD‐CP McPs). It was exhibited that the structural color of microparticles changed from green to blue in reverse replication. Afterward, the loading of GOD and CP nanodots rendered a visual color change of inverse opal. The visual change of microparticles at different stages and the records of corresponding spectrum were also shown in Figure S2, Supporting Information. Meanwhile, the microstructure of PhCs templates and inverse opal were studied via scanning electron microscope (SEM). It proved that the silica nanoparticles on the surface of the template were closely packed in the form of hexagonal packing, and this packing manner was also applicative to inside nanoparticles according to Figure 2c i‐ii. After hydrogel filling, it could be seen clearly that the space between the nanoparticles was filled with the polymerized hydrogel (Figure 2c iii), which indicated that the hydrogel solution dispersed well in the template nanochannel. Due to the closed packing of nanoparticles in the PhCs template, the hydrogel particles owned a highly ordered three‐dimensional porous structure after silica nanoparticles etching (Figure 2c iv). Besides, the nanopores of the inverse opal scaffold are highly dependent on the size of silica nanoparticle and bring about more enzyme binding sites.

**Figure 3:**
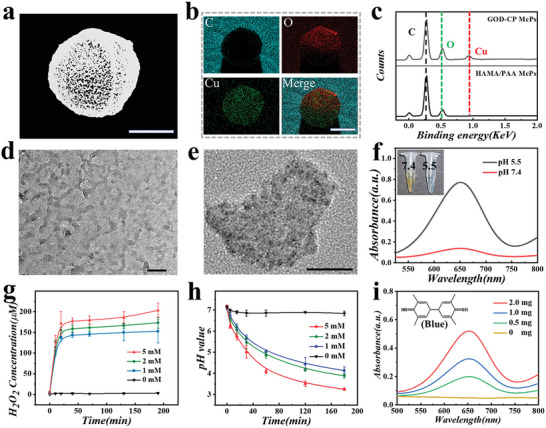
Characterization of composite microparticles. a‐b) Element mapping of composite microparticles. (c) Elements analysis of HAMA/PAA and GOD‐CP McPs. d‐e) The representative TEM images of CP nanodots. f) Catalytic properties of CP nanodots under different pH conditions in TMB and H_2_O_2_ solutions. g) H_2_O_2_ generation and h) pH decrease arising from the reaction between composite microparticles and different concentrations of glucose. i) Absorbance of TMB aqueous solution treated with different amounts of composite microparticles. Scale bars are 50 µm in (a, b), 20 nm in (d), and 10 nm in (e).

Besides, **Figure** **3**a–c displayed element mapping and analysis of composite microparticles. Results distinctly verified that such microparticles possessed abundant elements of C, O, and Cu, exhibiting efficient CP nanodots loading. To stably carry CP nanodots, polyvinyl pyrrolidone (PVP), acted as a stabilizer, was introduced. According to transmission electron microscopy (TEM) images, sizes of the formed PVP‐coated CP nanodots concentrated at 0–20 nm (Figure 3d‐e). Different from the blue copper‐ion solution, the dispersion of PVP‐coated CP nanodots was brownish‐yellow. In Figure S3a, Supporting Information, the UV‐vis spectrum of coated CP solution displayed its absorption in a wide wavelength range. These results have shown that the synthesized CP nanodots featured tiny sizes and well dispersion. Of note, such nanodots were sensitive to environmental pH. To investigate their pH responsiveness, PVP‐coated CP nanodots were collected by centrifuging and then dispersed in two PBS buffer solutions with pH value of 7.4 and 5.5, respectively. After 24 h for static standing, because of the decomposition of such nanodots, the color of solution with pH 5.5 changed from tan to blue. After that, these two solutions were added into 3,3″,5,5″‐Tetramethylbenzidine (TMB) solution in the existence of H_2_O_2_, respectively. As shown in Figure 3f, the ROS generated by catalysis was colored by TMB and had a distinct absorption at 652 nm. We noticed that the TMB solutions only treated with H_2_O_2_ or decomposed CP nanodots did not exhibit a significant generation of ROS (Figure S3b, Supporting Information).

It was necessary to study the performance of immobilized enzyme. As a brilliant glucose scavenger, GOD efficiently catalyzed glucose into gluconic acid and H_2_O_2_. It was clear that the concentration of H_2_O_2_ rapidly reached a high level in the existence of glucose and composite microparticles (Figure 3g). With the increase of glucose concentration, the reaction rate was enhanced in the experimental range. Meanwhile, the fast reduction in pH value caused by GOD catalysis reflected high bioactivity of immobilized enzyme (Figure 3h). For composite microparticles with GOD immobilization and CP nanodots loading, glucose could be quickly utilized, which caused the production of H_2_O_2_ and change of pH value. Then, hydroxyl radicals (●OH) were generated by a Fenton reaction in the presence of H_2_O_2_ and decomposed CP nanodots. While in the same concentration of glucose, the number of composite microparticles directly determined the rate of product formation (Figure 3i). Of note, we also investigated chemodynamic properties of different components, and the result of ROS formation was shown in Figure S4, Supporting Information. It was found that the cascade reaction between immobilized GOD and acid‐decomposed CP nanodots greatly enhanced the performance of ROS generation.

**Figure 4:**
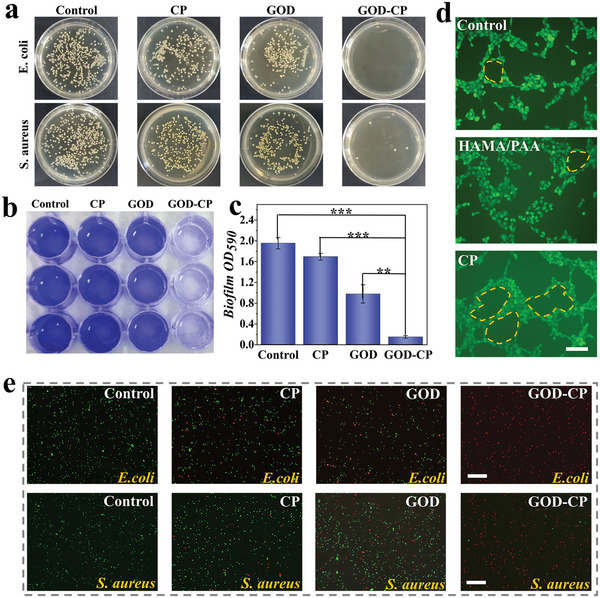
Antibacterial and stimulating angiogenesis properties of GOD McPs. a) Images of bacterial colonies on the culture plate after corresponding treatment. b) Images of antibiofilm performance in different groups (Blue: crystal violet). c) Analysis of absorption at 590 nm of stained biofilm in corresponding groups (*n* = 6). Data are shown as mean ± SD. d) Corresponding images of tube formation (Green: calcein). e) Fluorescent staining of live/dead *E. coli* and *S. aureus* after corresponding treatments (Green: SYTO; Red: PI). Scale bars represent 100 µm in (d) and 50 µm in (e). *p < 0.05, **p < 0.01, ***p < 0.001.

Such composite microparticles consumed glucose and generated ROS, thereby reducing glucose level and killing bacteria. To investigate the antibacterial properties of these composite microparticles, *Escherichia coli* (*E. coli*) and *Staphylococcus aureus* (*S. aureus*) were chosen to construct bacterial model. As illustrated in **Figure** **4**a, the GOD‐CP McPs group showed fewer bacterial colonies than other groups, exhibiting excellent antibacterial effects both for *E. coli* and *S. aureus*. The result was also supported by the corresponding bacterial live/death staining images recorded in Figure 4e. In addition, bacterial biofilm made great obstacles to the penetration and effect of drugs, bringing a certain drug resistance. Hence, we also explored the destruction effect of microparticles on the biofilm, as shown in Figure 4b‐c. The staining of crystal violet showed that the GOD‐CP McPs produced sufficient ROS to destroy bacterial biofilm induced by *S. aureus*. Compared to traditional antibiotic therapy, this ROS‐generation method exhibited effective antibacterial properties, and avoided the negative effects brought by the abuse of antibiotics, such as the establishment of drug‐resistant bacteria, the effects of metabolic function, etc. Notably, the ROS generated only at the beginning of the infection with the existence of wound glucose. Copper elements not only participated in the generation of ROS but could also stimulate angiogenesis by speeding up the recruiting of vascular endothelial growth factor. In contrast to the control and blank HAMA/PAA McPs groups, the experimental group with decomposed CP nanodots had shown a better angiogenesis effect in Figure 4d. These results demonstrated that GOD‐CP McPs possessed preeminent chemodynamic properties and angiogenesis capacity.

**Figure 5:**
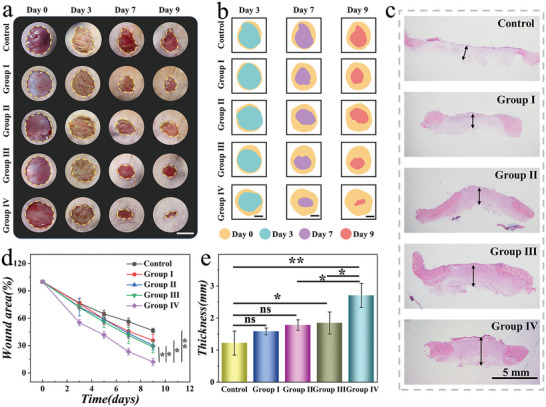
Evaluation of GOD‐CP McPs on the infectious wounds of diabetic rats. a) Photograph of wound states in different groups within nine days. b) Diagram of relative changes in wound area within nine days. c) H&E staining images of skin tissues. d) Statistic chart of the changes of wound area within treatment (*n* = 6, biologically independent samples). e) Statistic analysis of the thickness of wound tissues on Day 9 (*n* = 6, biologically independent samples). Scale bars are 10 mm in (a), 5 mm in (b), and 5 mm in (c). Data are sh as mean ± SD, NS: no significant, *p < 0.05, **p < 0.01.

To confirm the clinical value of such GOD‐CP McPs, the diabetic acute wound was built as the experimental model. First, the degradation and cytotoxicity were carried out to verify the biocompatibility and utilization efficiency of the microparticles. As shown in Figure S5, Supporting Information, the hydrogel microparticles possessed an outstanding degradation performance in buffer solution. Besides, inverse opal material composed of HAMA and AA had an almost negligible effect on cell growth over three days, as shown in Figure S6, Supporting Information. In the wound healing assay, Sprague−Dawley rats had type I diabetes, suffering from a round wound on their back. These rats were randomly divided into five groups, and different treatments were applied. Rats in the control group received PBS buffer to soak the wound. In contrast, the other four groups were treated with HAMA/PAA carrier (Group I), HAMA/PAA carrier with CP nanodots loading (Group II), GOD McPs (Group III), and GOD‐CP McPs (Group IV), respectively. The status of wounds in different groups was recorded for further analysis, as shown in **Figure** **5**a,b. It was observed that the Group IV owned a superior area closure compared to other groups. Compared with other treatment systems proposed in literature, our experimental group also showed a satisfactory wound treatment effect.

**Figure 6:**
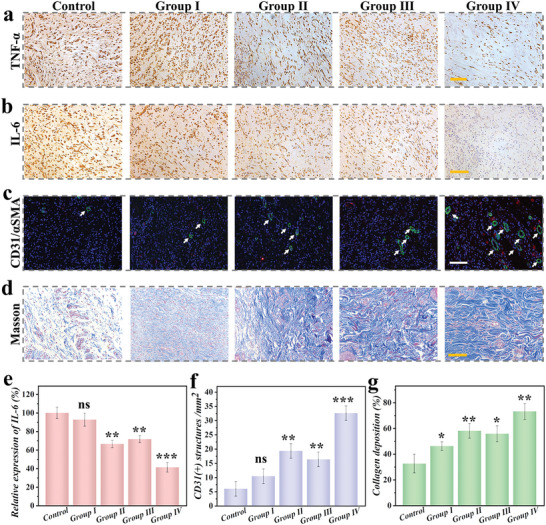
Biological mechanism of composite McPs on the diabetic wound. a) Immunohistochemical staining of TNF‐*α* expression and b) IL‐6 expression on Day 9. c) Images of immunofluorescence staining on Day 9. (Green: *α*SMA; Red: CD31). d) Images of Masson staining in different groups on Day9. (Blue: collagen). e) Statistic analysis of the relative expression of IL‐6,(f) CD31(+) structures and (g) collagen deposition on Day 9. Scale bars are all 100 µm. (*n* = 6, biologically independent samples). Data are shown as mean ± SD. NS: no significant, * *p* < 0.05, ** *p* < 0.01, ***p < 0.001.

To explore the states of wound tissues, hematoxylin‐eosin (H&E) staining was performed. As shown in Figure 5c, the wounds in Group IV had a thicker regenerated tissue, while that in the control group was the thinnest. Based on the statistics of wound area and re‐epithelialization thickness (Figure 5d,e), Group IV showed a smaller wound area and more complete regeneration tissue by comparison to Day 9. Except for H&E staining, other characterization methods were also carried out, including immunofluorescent staining, Masson staining, as well as immunohistochemical staining (**Figure** **6**). IL‐6 and TNF‐*α* were two important indicators to investigate the inflammatory status. Compared to other groups, Group IV displayed less expression of these factors, which confirmed the distinctive bactericidal effect. Additionally, blood vessels are important channels beneficial for tissue regeneration. The immunofluorescent staining results exhibited that the group treated with GOD‐CP microparticles showed a greater vessel density, which was attributed to the decomposition of CP nanodots (Figure 6c,f). Besides, the deposition and arrangement of collagen provide an effective recovery environment for tissue regeneration. As shown in Figure 6c,g, collagens in Group IV exhibited a high degree of directional alignment in Masson staining, which proved to improve wound microenvironment and healing status. Notably, another feature of microparticles was the sensitive structural color sensing based on pH response. As shown in Figure S7, Supporting Information, the same microparticles displayed different colors when the environment was under changed pH conditions. Specifically, the spectrum of microparticles gradually exhibited a trend of blue shift when pH value decreased. Therefore, the catalytic process of glucose and microenvironment of the wound could be evaluated by visual observation and spectral measurement of microparticles. This nonchemical real‐time monitoring strategy transforms biological signals into visual optical signals, thus avoiding the contamination caused by the use of dyes. These results demonstrated that such enzyme cascade structural color hydrogel microparticles possessed practical values in wound management.

## 3 Conclusion

In this paper, we have presented the cascade catalytic inverse opal hydrogel system with GOD immobilization and CP nanodots loading. The inverse opal, structured by HAMA and pH‐responsive acrylic acid (AA), owned abundant modifiable functional groups. Through chemical modification and physical doping, the inverse opal hydrogel beads realized loading of enzymes and a metal salt. Once these composite beads were applied to the wound site, the hyperglycemia environment served as a hydrogen peroxide (H_2_O_2_) generator through GOD catalysis instead of a nutrient supplier of bacteria. Meanwhile, an acidic wound environment triggered the decomposition of CP in the microparticles, which would effectively further catalyzed H_2_O_2_ to generate antibacterial reaction oxygen species (ROS). It was demonstrated that the resultant composite microparticles could effectively improve the performance of sterilization in vivo experiments. These results exhibited that such microparticles possessed enhanced chemo‐dynamism properties and would find ideal applications in antibacterial and wound healing fields.

## 4 Experimental Section

### Materials

Glucose oxidase (GOD), acrylic acid (AA), Glucose, octadecyl trichlorosilane (OTS), and polyvinyl pyrrolidone (PVP) were obtained from Sigma‐Aldrich (St. Louis, MO, USA). Hydrofluoric acid, Copper chloride (CuCl_2_), and sodium hydroxide (NaOH) were brought from Aladdin Industrial Corporation. HAMA was synthesized according to previous work. Hydrochloric acid (HCl, 12 mol), potassium dihydrogen phosphate dodecahydrate (KH_2_PO_4_.12H_2_O), potassium dihydrogen phosphate (K_2_PO_4_), n‐hexane, and phenol were brought from Sinopharm Chemical Reagent. Hydrogen Peroxide Assay Kit was brought from Beyotime Biotechnology in Shanghai. All reagents were analytical grade or higher and all reagents were used as received. The water was fabricated by Milli‐Q Plus 185 water purification system (Millipore, Bedford, MA).

### Generation of Microfluid Chip

The microfluidic device was composed of two capillary tubes which were coaxially assembled on one slide. The outer diameter of the capillary tubes was 1000 µm and the inner diameter was 800 µm. Before use, the tubes were drained by hydrophobic OTS for several minutes. The inner diameter of the pointed end of the tube that the inner phase solution flowed through was tapered to reach ≈250 µm. Then, such tubes were coaxially assembled and the needle was combined with glue at the joint of two capillary tubes.

### Fabrication of Silica Colloidal Crystal Template

The silica colloidal crystal particles were generated by the microfluidic device above. At first, the concentration of the nanoparticles with different sizes should be adjusted to ≈20% (w/v). Then, such solution was pumped into the inner capillary tube. Meanwhile, silicone oil of 50 CS served as the outer phase. The volume of the oil phase was set as 5 mL and the flow rate was 3 mL/h. The volume of the aqueous phase was set as 1 mL and the flow rate was 0.5 mL/h. The nanoparticle solution was dispersed into small droplets in the oil phase and was collected in high‐viscosity silicone oil. After that, these droplets were transferred to an oven at 75 °C for 10 h. In the process, the nanoparticles could self‐assemble orderly with the evaporation of water. Ten hours later, the silica crystal beads were washed with n‐hexane softly. In the end, the beads were calcined at 800 °C for 6 h. Then, the colloidal crystal could obtain better mechanical strength. The reflection spectrum of colloidal crystal was measured by a metallographic microscope (OLYMPUS BX51). The microstructure was observed by scanning electron microscopy (SEM, Hitachi, S300N).

### Fabrication of HAMA

The concentration of hyaluronic acid (HA) in solution was adjusted to 2%. Under stirring, 15 mL of MA was dropwise added to such solution. To fully react, the pH value of the reaction solution was maintained ≈8. After 24 h, plenty of alcohol was used to precipitate, and then the precipitate was suspended in pure water. Subsequently, the resuspension was dialyzed with pure water for three days. Finally, HAMA was obtained by freeze‐drying.

### Preparation of Porous Inverse Opal Hydrogel Particles

The hydrogel mainly consisted of three components including 10% HAMA, 10% AA, and 1% 2‐hydroxy‐2‐methylpropiophenone (HMPP). The colloidal crystal particles were immersed in the hydrogel solution for about half an hour. Because of the capillary force, the hydrogel can be filled in the gap in the template. Then ultraviolet (UV) was used to polymerize the hydrogel, and the hybrid particles could be stripped from the gel and immersed in the 4% hydrofluoric acid for 6 h to remove the silica nanoparticles. After being cleaned, the prepared opal hydrogel microparticles were vacuum freeze‐dried for subsequent use.

### Synthesis of CP Nanodots

In brief, PVP served as a stabilizer to add to the Cupric chloride (CuCl_2_) solution. Then, the alkaline solution and H_2_O_2_ was added in the system in turn. After a period of reaction, the system was filtered and washed to obtain CP nanodots. The characterization of such nanodots was performed by high‐resolution transmission electron microscopy (FEI Talos).

### Properties of CP Nanodots

To verify the pH‐triggered decomposition of CP nanodots, the centrifuged precipitate was resuspended in the PBS solutions with different pH values. After 24 h of static standing, the state and color of nanodot solutions with pH 7.4 and 5.5 were recorded. Meanwhile, their respective catalytic capacities were studied by TMB color reaction. To compare the generation of ROS species, the TMB solution was divided into three groups, which were treated with decomposition CP plus H_2_O_2_, decomposition CP, and H_2_O_2,_ respectively. Afterwards, the spectra were detected from 500 nm to 800 nm.

### Fabrication of Immobilized Enzyme and Composite McPs

First, the dried hydrogel microparticles were immersed in MES buffer (pH 6.0) solution with 0.055 g of EDC and 0.035 g of NHS. This process would sustain 30 min at 37 °C. After the reaction, the inverse opal hydrogel particles were washed with PBS (PH 5.6) to remove residual glutaraldehyde. GOD was weighed and dissolved in phosphoric acid buffer until its concentration was 10 mg mL^−1^. The inverse opal hydrogel particles were immersed in the enzyme solution for 12 h at 4 °C and mixed well every other hour. Through this process, GOD could be evenly immobilized on the inverse opal hydrogel particles. Then, GOD McPs were immersed in CP nanodots solution to achieve payload.

### Detection of Enzymatic Activities of GOD‐Immobilized Inverse Opal Hydrogel

GOD‐immobilized McPs were put into a glucose solution with different concentrations of 0, 1, 2, and 5 mM. Subsequently, a pH meter (SX 620) was used to measure the pH value of reaction solution at different points. Meanwhile, the generation of H_2_O_2_ was detected by the assay kit.

### Chemodynamic Activity of the Composite McPs

Different amounts of composite microparticles were added in 96‐well plates with 190 µL of buffer solution. Then, the combined solution was shaken at 37 °C in the dark for 30 min. After that, TMB (1 µL, 0.1 M) and glucose (10 µL, 0.1 M) was supplemented in the resulting system and incubated at 37°C for 3 h. At last, TMB absorbance of 652 nm was detected to measure the production of hydroxyl radicals (•OH).

### Antibacterial Assay In Vitro

To study the antibacterial ability of composite hydrogel microparticles, *Escherichia coli* (*E. coli*) and *Staphylococcus aureus* (*S. aureus*) were chosen to build bacterial models. At first, the bacteria suspension was centrifuged at 1500 rpm. Then, the buffer solution containing 2% glucose was used to disperse precipitate until the turbidity value of the suspension reached 0.5. The test was divided into four groups, including a) control, b) CP, c) GOD, and d) GOD‐CP. After 12 h incubation, the supernatant was pipetted for further plate culture. The treated plate was placed into the shaking incubator at 37 °C for 12 h. Subsequently, images of single colonies on the plate were recorded for comparison. Besides, the bacterial suspension was stained by a live/dead staining kit (SYTO/PI) and photographed using an inverted fluorescence microscope (Axio Observer A1).

### Antibiofilm Study In Vitro

In this section, *S. aureus* was used to establish a biofilm model. First, the same amount of culture medium and bacterium were added to each well of the 24‐well plate. Of note, the culture medium included 5% glucose. Subsequently, the samples received different treatments, which were divided into four groups, including a) control, b) CP, c) GOD, and d) GOD‐CP. The crystal violet staining was carried out 12 h later to estimate the anti‐biofilm effect. Specially, each well dripped 200 µL of crystal violet dye (1%) for 5 min. After removing the excess dye, the culture system was dried at a temperature of 37 °C. Finally, 200 µL of acetic acid (33%) was added in the well reaction for 30 min. The absorbance at 590 nm was determined via UV–vis–NIR spectroscopy.

### Biocompatibility Test In Vitro

Biofriendly materials were subjected to MTT assays according to previous works. In short, 3T3 cells were incubated in 48‐well plate with DMEM culture medium. There were two groups diving in this experiment including control group with no treatment and experimental group with HAMA/PAA hydrogel added. After incubation for 24 h, 48 h, and 72 h, 10% MTT of final concentration was added to the orifice plate. After another 4 h, the composite solution was aspirated from each well. Subsequently, DMSO was added to dissolve the purple compounds, and the OD value of such solution was measured by a microplate reader. Finally, 3T3 cells stained by calcein‐AM and propidium iodide (PI) were also imaged by fluorescent microscope.

### Wound Healing Assay

All the animal experiments were approved by the Animal Ethics Committee of the Wenzhou Institute (University of Chinese Academy of Sciences), and the approval number was WIUCAS22080803. The Sprague−Dawley rats (250 g) were purchased from Qinglongshan Animal Farm. To induce diabetes, each rat was intraperitoneally injecte 1% (w/v) streptozotocin (STZ), and the dosage of STZ was 75 mg kg^−1^. Afterward, the blood glucose level was monitored every day to ensure the success of fabricating the diabetic model.

After a week, the induced rats were treated with back skin circumcision and bacterial suspension was added on the wound surface. Then, these experimental subjects were stochastically divided into five groups and each group was suffered from corresponding treatments. The first group, also named the control group, was only rinsed with PBS buffer solution. Another four groups were treated with HAMA/PAA carrier (Group I), HAMA/PAA carrier with CP loading (Group II), GOD McPs (Group III), and GOD‐CP McPs (Group IV), respectively. Wound images of different groups were recorded on Day 0, 3, 7, and 9. On the last day of treatment, all rats were euthanized. Finally, the granulation tissue from the wound was cut out, and preserved in neutral formaldehyde. In the end, the tissue was embedded in paraffin and sectioned for further staining and analysis.

### Statistical Analysis

Results were exhibited as the means ± standard deviation (SD). Statistical analysis was carried out using Student's *t*‐test or one‐way ANOVA followed by Tukey's post‐hoc test to determine the degree of significance by the software of Origin 2022. Statistical significance was defined as NS: no significant, *p < 0.05, **p < 0.01, ***p < 0.001.

## Conflict of Interest

The authors declare no conflict of interest.

## Supporting information

Supporting Information

## Data Availability

The data that support the findings of this study are available from the corresponding author upon reasonable request.
