# Supplementary material for: Biomimetic Enzyme Cascade Structural Color Hydrogel Microparticles for Diabetic Wound Healing Management
Source: Adv Sci (Weinh). 2023 Mar 22;10(14):2206900. doi: 10.1002/advs.202206900 (PMC10190660; doi:10.1002/advs.202206900)
Supplement: Supplementary file 1 — Supporting Information [file ADVS-10-2206900-s001.pdf]

ADVANCED SCIENCE Open Access

Supporting Information

for *Adv. Sci.*, DOI 10.1002/advs.202206900

Biomimetic Enzyme Cascade Structural Color Hydrogel Microparticles for Diabetic Wound Healing Management

Li Wang, Guopu Chen, Lu Fan, Hanxu Chen, Yuanjin Zhao, Ling Lu* and Luoran Shang**

Supporting Information

Biomimetic enzyme cascade structural color hydrogel microparticles for diabetic wound healing management

Li Wang, Guopu Chen, Lu Fan, Hanxu Chen, Luoran Shang, Ling Lu*, Yuanjin Zhao**

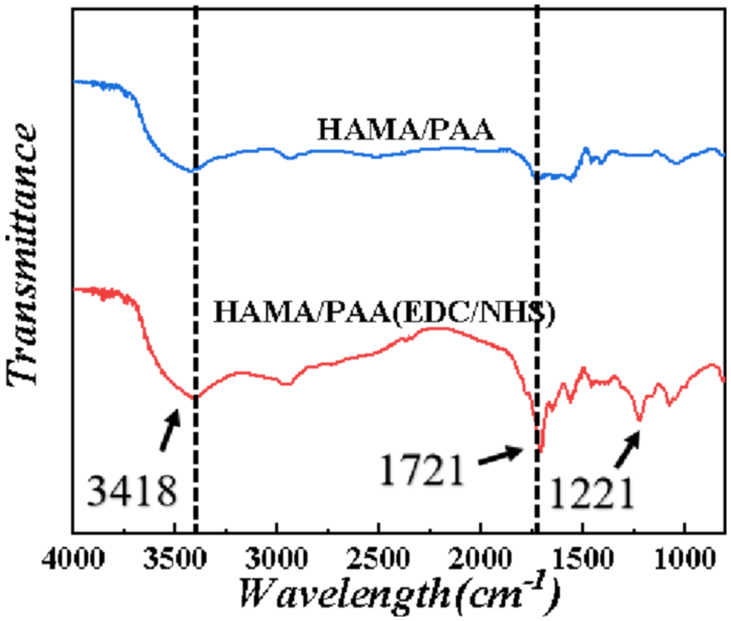

Figure S1. FT-IR spectra of hydrogel before and after activation of carboxyl groups.

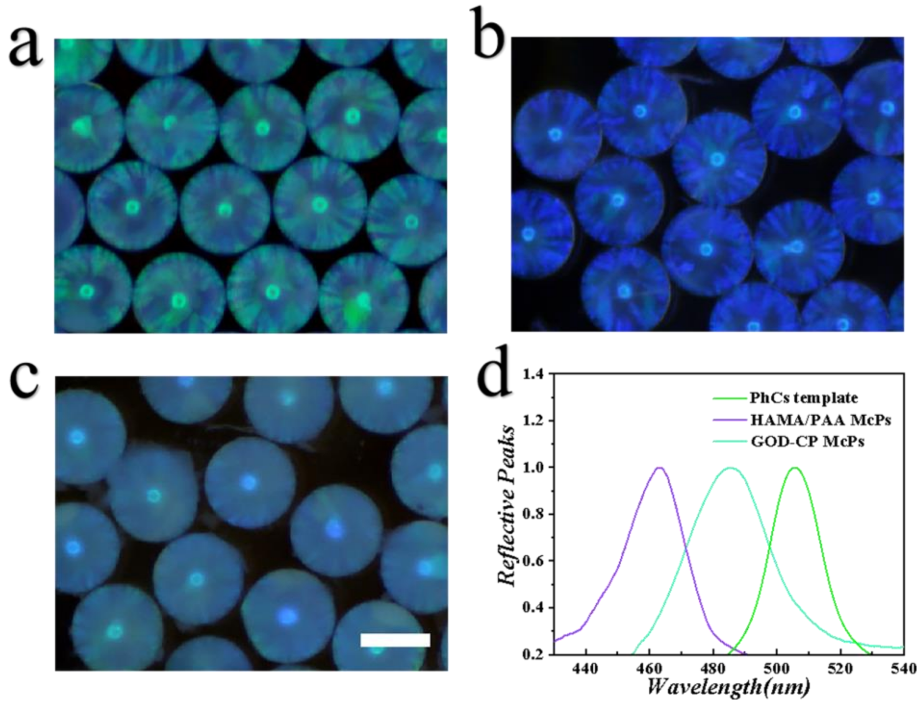

Figure S2. Images of different McPs and corresponding spectral peak changes. (a) PhCs templates; (b) HAMA-PAA McPs; (c) GOD-CP McPs. Scale bars are 200 nm.

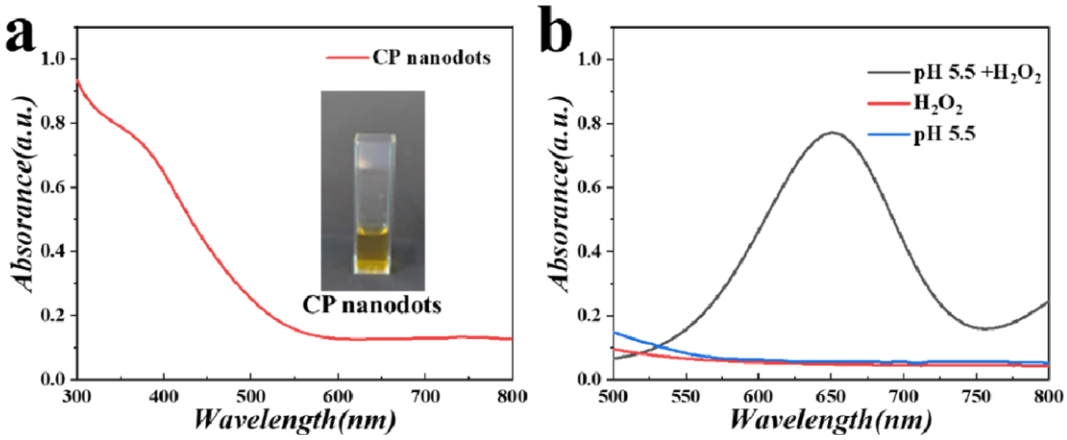

Figure S3. Characteristic of CP nanodots. (a) UV-vis area absorption spectrum of PVP-stabilized nanodots solution; (b) UV-vis spectra of TMB chromogenic solution treated with CP nanodots (pH 5.5) plus H_2O_2, H_2O_2, and CP nanodots alone.

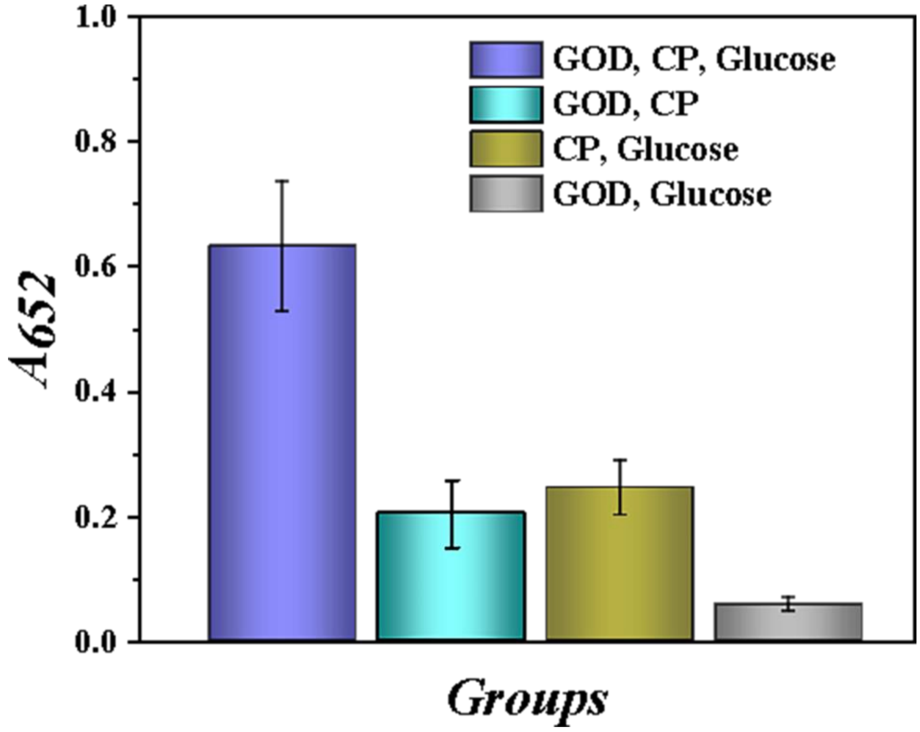

Figure S4. Statistic of ROS generated in different groups under different GSH concentration.

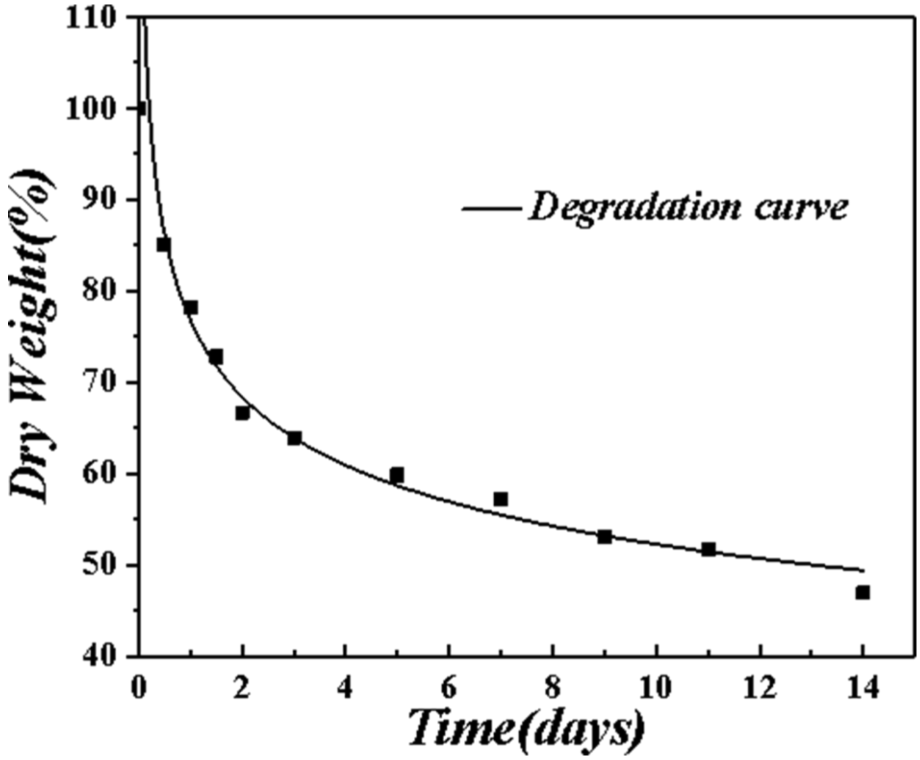

Figure S5. Degradation curve of composite HAMA/PAA McPs in 14 Days.

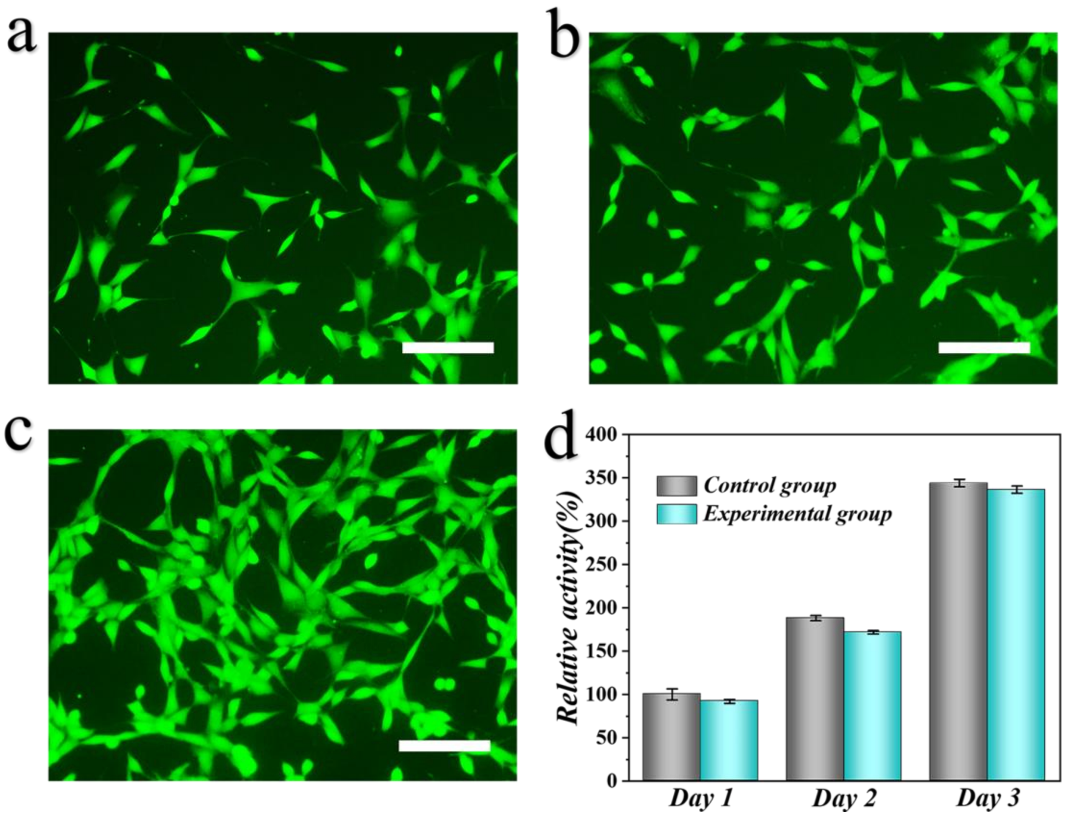

Figure S6. Cytotoxicity test in vitro. (a-c) The state of 3T3 cell cultured in experimental group on different days; (d) Relative activity of 3T3 cell in control and experimental group within three days. Scale bars are 50 μm.

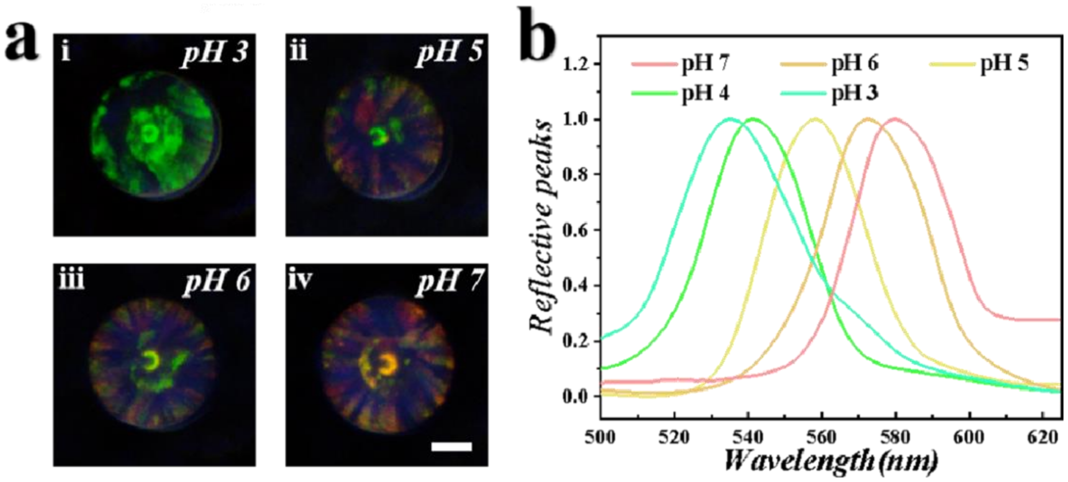

Figure S7. pH responsiveness of composite McPs in the buffer solution with different pH value and the corresponding spectrum changes. Scale bar is 100 μm in (a).
